# Editorial: Biofabricated materials for tissue engineering

**DOI:** 10.3389/fbioe.2024.1407811

**Published:** 2024-05-08

**Authors:** Hilal Turkoglu Sasmazel, Oguzhan Gunduz, Murugan Ramalingam, Songul Ulag

**Affiliations:** ^1^ Metallurgical and Materials Engineering Department, School of Engineering, Atılım University, Ankara, Türkiye; ^2^ Center for Nanotechnology and Biomaterials Research, Marmara University, Istanbul, Türkiye; ^3^ Joint Research Laboratory (JRL) on Bioprinting and Advanced Pharma Development, A Joint Venture of TECNALIA and University of the Basque Country (UPV/EHU), Centro de investigación Lascaray ikergunea, Vitoria-Gasteiz, Spain; ^4^ NanoBioCel Group, Department of Pharmacy and Food Sciences, Faculty of Pharmacy, University of the Basque Country (UPV/EHU), Vitoria-Gasteiz, Spain; ^5^ IKERBASQUE, Basque Foundation for Science, Bilbao, Spain; ^6^ Bioaraba Health Research Institute, Vitoria-Gasteiz, Spain; ^7^ Biomedical Research Networking Centre in Bioengineering, Biomaterials and Nanomedicine (CIBER-BBN), Institute of Health Carlos III, Madrid, Spain; ^8^ Metallurgical and Materials Engineering Department, Marmara University, Istanbul, Türkiye

**Keywords:** biomaterials, biofabrication, tissue regeneration, tissue repair, tissue engineering

In the realm of regenerative medicine, biofabricated materials have emerged as a beacon of hope, offering unparalleled opportunities for tissue regeneration and repair. These materials, often derived from natural sources or engineered using cutting-edge technologies, hold immense potential to revolutionize healthcare by addressing crucial challenges in organ transplantation, wound healing, and tissue engineering.

To delve into the remarkable advancements in biofabricated materials and their transformative impact on the field of tissue regeneration Frontiers in Bioengineering and Biotechnology developed a Research Topic “Biofabricated Materials for Tissue Regeneration” with the Research Topic editors Dr. Turkoglu Sasmazel, Dr. Gunduz, Dr. Ramalingam, and the Research Topic coordinator Dr. Ulag in 2023–2024.

Four articles were published under this title.

Selsouli et al. optimized a tunable process for rapid production of calcium phosphate microparticles using a droplet-based microfluidic platform. They achieved adjusting synthesis parameters, such as precursor concentration, pH value, and aging time, and applying heat treatment and indicated that the synthesis and fabrication parameters of CaPs in their method can alter the microstructure and the degradation behavior of CaPs. Finally, their research highlighted the potential of the droplet microfluidic platform for engineering CaP microparticle biomaterials with fine-tuned properties.

Dogan et al. studied how the degree of methacrylate (MA), GelMA mass concentration, and cell density change mass transport properties. For this purpose, they encapsulated cells in gelatin methacryloyl bioinks impairs microscale diffusion properties. They introduced a fluorescent-microscopy-based method of biotransport testing with improved sensitivity compared to the traditional particle tracking methods in this study.

Rifai et al. provided an overview of the structure and mechanical properties of natural bone, the role of bone cells, the remodeling process, cytokines, and signaling pathways, causes of bone defects, and typical treatments and new tissue engineering strategies. In this review, they highlighted processes of selecting biomaterials, cells, and growth factors. Furthermore, they discussed innovative tissue-engineered models for cancer treatments, injectable stimuli gels, and other therapeutic drug delivery systems. Finally, they reviewed the current challenges and prospects of bone tissue engineering.

Fadilah et al. released a review titled Functionalised-Biomatrix for Wound Healing and Cutaneous Regeneration: Future Impactful Medical Products in Clinical Translation and Precision Medicine. They outlined the wound healing mechanisms employed by commercially available engineered skin replacements (ESS), emphasizing the need for a versatile and advanced ESS substitute. Additionally, this study examined the utilization of multifunctional bioscaffolds in wound healing scenarios, showcasing their effective biological functionality in both *in vitro* and *in vivo* animal models.

Despite significant progress, several challenges persist in the field of biofabricated materials for tissue regeneration. These include scalability for clinical translation, long-term stability of constructs, immune responses, and regulatory considerations. Addressing these challenges requires interdisciplinary collaboration, innovative manufacturing technologies, and rigorous preclinical and clinical evaluations.

Looking ahead, the future of biofabricated materials holds immense promise. Advancements in bioengineering, artificial intelligence, and regenerative medicine will drive the development of next-generation biomaterials with enhanced functionalities, bioactivity, and therapeutic efficacy. Moreover, the integration of bioprinting with patient-specific data, such as genomics and imaging, will enable precise customization of tissues and organs, ushering in a new era of personalized regenerative therapies.

Biofabricated materials represent a paradigm shift in tissue regeneration, offering tailored solutions for repairing and replacing damaged tissues and organs. The convergence of biomaterials science, nanotechnology, cell biology, and bioengineering is fueling rapid progress in this field. As researchers continue to innovate and collaborate, biofabricated materials hold the potential to address unmet medical needs, improve patient outcomes, and transform the landscape of healthcare in the years to come.

We would like to thank the authors for their contributions to this Research Topic on the Biofabricated Materials for Tissue Regeneration.

